# Decreased mitochondrial DNA content in blood samples of patients with stage I breast cancer

**DOI:** 10.1186/1471-2407-9-454

**Published:** 2009-12-21

**Authors:** Peng Xia, Han-Xiang An, Cheng-Xue Dang, Ramin Radpour, Corina Kohler, Emmanouil Fokas, Rita Engenhart-Cabillic, Wolfgang Holzgreve, Xiao Yan Zhong

**Affiliations:** 1 Department of Surgical Oncology, First Affiliated Hospital of Medical College, Xi'an Jiaotong University, 710061 Xi'an, PR China; 2 Laboratory for Prenatal Medicine and Gynecological Oncology, Women's Hospital and Department of Biomedicine, University of Basel, Switzerland; 3 Department of Radiotherapy and Radiation Oncology, Philipps-University Marburg, Baldingerstr. D-35043 Marburg, Germany; 4 Medical Center, Abert-Ludwigs-University-Freiburg, Germany

## Abstract

**Background:**

Alterations of mitochondrial DNA (mtDNA) have been implicated in carcinogenesis. We developed an accurate multiplex quantitative real-time PCR for synchronized determination of mtDNA and nuclear DNA (nDNA). We sought to investigate whether mtDNA content in the peripheral blood of breast cancer patients is associated with clinical and pathological parameters.

**Methods:**

Peripheral blood samples were collected from 60 patients with breast cancer and 51 age-matched healthy individuals as control. DNA was extracted from peripheral blood for the quantification of mtDNA and nDNA, using a one-step multiplex real-time PCR. A FAM labeled MGB probe and primers were used to amplify the mtDNA sequence of the ATP 8 gene, and a VIC labeled MGB probe and primers were employed to amplify the glyceraldehyde-3-phosphate-dehydrogenase gene. mtDNA content was correlated with tumor stage, menstruation status, and age of patients as well as lymph node status and the expression of estrogen receptor (ER), progesterone receptor (PR) and Her-2/neu protein.

**Results:**

The content of mtDNA in stage I breast cancer patients was significantly lower than in other stages (overall P = 0.023). Reduced mtDNA was found often in post menopausal cancer group (P = 0.024). No difference in mtDNA content, in regards to age (p = 0.564), lymph node involvement (p = 0.673), ER (p = 0.877), PR (p = 0.763), and Her-2/neu expression (p = 0.335), was observed.

**Conclusion:**

Early detection of breast cancer has proved difficult and current detection methods are inadequate. In the present study, decreased mtDNA content in the peripheral blood of patients with breast cancer was strongly associated with stage I. The use of mtDNA may have diagnostic value and further studies are required to validate it as a potential biomarker for early detection of breast cancer.

## Background

More than 75 years since Warburg described how tumor cells avidly consume glucose and produce lactic acid under aerobic conditions, it still remains unclear how this metabolic shift provides tumor cells with a growth advantage[1,2]. Recent evidence has shown that tumor cells adapt their metabolism to the microenvironment by suppressing mitochondrial function rather than increasing glycolysis [3]. In patients with mitochondrial disease, mitochondrial function is vulnerable to damages due to deletions, mutations or replication abnormalities of mitochondrial DNA (mtDNA) resulting in energy depletion and increased susceptibility to apoptosis [4]. Additionally, mtDNA alterations are correlated with various cancer types, suggesting that the mitochondrial genome may be a critical contributing factor in carcinogenesis. mtDNA content has been implicated as a potential biomarker for several cancer types [5]. Decreased mtDNA content had been reported for renal [6], gastric [7], breast [5,8], previously-treated head and neck [9], ovarian [10] and hepatic cancer [11-13]. In contrast, several studies have revealed an increased mtDNA content in prostate [14], untreated head and neck [15], thyroid [5], endometrial [16], and pancreatic cancer [17]. Interestingly, mtDNA alterations were also detected in bodily fluids, suggesting that mtDNA changes might serve as sensitive early biomarker for non-invasive detection of several types of solid cancer including breast cancer [18].

Similarly to previous reports [5], we have shown that mtDNA content was decreased in 82% of cancerous breast tissues, as compared with the normal ones[19]. However, to our best knowledge, no data exist regarding mtDNA content in peripheral blood of breast cancer patients and its correlation with clinical-pathological parameters. In the present study, we measured mtDNA content from peripheral blood samples of patients with breast cancer using a novel multiplex quantitative real-time PCR, as previously described [20]. The association between peripheral blood mtDNA content and clinical-and pathological parameters was analyzed and compared with the healthy donors.

## Methods

### Sample collection

Blood samples from 60 patients with breast cancer were taken before primary surgery. All patients were diagnosed between 2005 and 2007 and underwent surgery at the First Affiliated Hospital of Medical School of Xi'an Jiao Tong University of China. 51 control samples were randomly selected among individuals visiting hospitals for regular health checks. All patients gave informed consent for retention and analysis their blood for research purpose according to institutional guidelines and the study was approved by the research ethics committee of the Medical School of Xi'an Jiao Tong University, China. Tumors were staged according to the TNM classification (Union Internationale Contre le Cancer, UICC). None of patients received neoadjuvant treatment or have distant metastases as the time of primary surgery. Hematoxylin and eosin staining was used for routine pathological evaluation. Expression of estrogen receptor (ER), progesterone receptor (PR) and Her-2/neu protein was determined by immunohistochemistry. Clinical-pathological data (age, histological grade, tumor size, lymph node status, ER-, PR-, and Her-2/neu expression) are summarized in Table 1. The median ages of the cancer and the control group were 49.5 ± 1.6 years and 51.0 ± 2.0 years, respectively.

**Table 1:** Patient's data and correlation between levels of mtDNA in peripheral blood of patients with breast cancer and clinical-pathological parameters

Variable	Number	mtDNA content Median (Range)	P value
Age (years)			
<50	30	525.96 (0.47 - 35241.80)	*0.564
≥50	30	660.59 (0.39 - 27175.14)	
Menopausal status			
pre	37	1365.30 (0.47 - 35241.80)	*0.024
post	23	262.29 (0.39 - 5166.60)	
Lymph-node status			
negative	29	668.60 (4.23 - 27175.14)	*0.673
positive	31	436.55 (0.39 - 35241.80)	
Stage			
I	20	196.94 (0.47 - 6841.04)	**0.023
II	20	1656.18 (10.89 - 35241.80)	
III-IV	20	572.71 (0.39 - 4956.14)	
ER			
positive	29	657.11 (0.39 - 35241.80)	*0.877
negative	31	376.11 (0.47 - 27175.14)	
PR			
positive	36	654.85 (4.23 - 27175.14)	*0.763
negative	24	669.09 (0.39 - 35241.80)	
Her2/neu protein			
positive	32	242.96 (0.39 - 35241.80)	*0.335
negative	28	824.78 (10.89 - 27175.14)	

*P values are obtained by using the Mann-Whitney U test. ** P values are obtained by using one-way ANOVA on the rank followed by the Tukey test.

### DNA isolation and multiplex quantitative real-time PCR

DNA was subsequently isolated from whole blood by phenol-chloroform extraction and ethanol precipitation [21]. The DNA samples were divided into aliquots of 100 μl and were stored in -80°C. The ABI PRISM 7000 Sequence Detection System (Applied Biosystems, ABI) was used to amplify the GAPDH housekeeping gene and the mitochondrial DNA encoded ATPase (MTATP) 8 gene. Primers and probes used for detection of GAPDH and MTATP 8 gene sequences were: GAPDH (forward): 5'-CCC CAC ACA CAT GCA CTT ACC; (reverse): 5'-CCT AGT CCC AGG GCT TTG ATT; probe 5'-(MGB)-TAG GAA GGA CAG GCA AC (VIC). Mitochondrial DNA (forward): 5'-AAT ATT AAA CAC AAA CTA CCA CCT ACC; (reverse): 5'-TGG TTC TCA GGG TTT GTT ATA; probe: 5'-(MGB)-CCT CAC CAA AGC CCA TA (FAM). The real-time RT-PCR was carried out in 25 μl of total reaction volume containing 5 μl (40 ng) of DNA, 12.5 μl of TaqMan^® ^Universal PCR Master Mix, 4 primers and 2 probes using a 2 minute incubation at 50°C, followed by an initial denature step at 95°C for 10 minutes and 40 cycles of 1 minute at 60°C and 15 seconds at 95°C. All samples were analyzed in triplicate. To determine the quantities of mtDNA and nDNA present in blood samples, the average threshold cycle number (Ct) values of the nDNA and mtDNA were obtained from each case. The level of mtDNA was calculated using the delta Ct (ΔCt) of average Ct of mtDNA and nDNA (ΔCt = CtmtDNA-CtnDNA) in the same well as an exponent of 2 (2^ΔCt^).

### Statistical analysis

Contents of mtDNA and nDNA are given as the median, the range and the fold difference. Receiver operating characteristic (ROC) curves were generated to estimate the sensitivity and specificity of mtDNA content. The Shapiro-Wilk test was used to analyze the data distribution. It showed that our data set was not normally distributed (p = 0.000 and 0.010 for the mtDNA- and the GAPDH assay, respectively). The two-sample Kolmogorov-Smirnov (K-S) test was used to compare the content of mtDNA in blood between the control- and breast cancer group. The Spearman's rho analysis was applied to analyze the relationship of mtDNA content in peripheral blood between the two groups. The Mann-Whitney U Test and Kruskal-Wallis test were employed to compare mtDNA content with clinical-pathological parameters in breast cancer group. We converted the mtDNA content values to ranks, and then did one-way ANOVA on the ranks in order to use the Tukey post-hoc procedure for the pairwise comparions of mtDNA content in relation to tumor stage. Differences were considered significant when p < 0.05. All statistical analyses were performed using the SPSS version 15.0 software (SPSS Inc., Chicago, IL).

## Results

### Measurements of the efficiency of multiplex Assays

The specificity of the assay for detecting the mtDNA was assessed using the mtDNA depleted 143b rho0cell line. No positive amplification of nucleus-embedded mtDNA sequences was obtained from the cell line using the assay for mtDNA (data not shown). To determine the quantities of mtDNA and nDNA present in blood samples, standard dilution curves were generated from a human genomic DNA with known concentrations, ranging from 3.125 × 10^4 ^to 10 pg/μl (including 31250, 6250, 1250, 250, 50 and 10 pg/μl). We used experimental serial dilutions to examine the amplification efficiencies for the MTAPT-8-gene amplicon (79 bp) for representing total mtDNA, and the GAPDH-gene amplicon (97 bp) for representing total nDNA. The amplifications of mtDNA and nDNA on serial dilutions showed a good correlation with comparable efficiencies of close to 99%.

### Co-extraction of mtDNA and nDNA from peripheral blood samples of breast cancer patients

The average Ct values of GAPDH ranged from 20.16 to 24.71 in blood samples of patients with breast cancer and from 21.73 to 25.73 in whole blood samples of the control group, respectively. The average Ct values of MTATP 8 gene ranged from 10.65 to 21.54 in blood samples of patients with breast cancer and from 11.78 to 14.88 in whole blood samples of the control group, respectively. In breast cancer patients, mtDNA content was higher than nDNA (Table 2).

**Table 2:** Level of mtDNA content in peripheral blood of the control- and breast cancer groups

	Normal N = 50	Breast Cancer N = 60	Change (ΔΔ Ct)	P value
ΔCt (Ct_nDNA _- Ct_mtDNA_)				
Median	10.43	9.36		
Range	6.07-12.03	-1.37-15.11	1.07	
mtDNA content				
Median	1374.79	654.85		*p = 0.009
Range	66.95-4182.07	0.39-35241.80	2.10	
Correlation				
(Ct_nDNA _vs Ct_mtDNA_)	r = 0.496 **p = 0.000	r = 0.148 **p = 0.259		

* Kolmogorov-Smirnov (K-S) test; ** Spearman's rho test.

**Figure 1:**
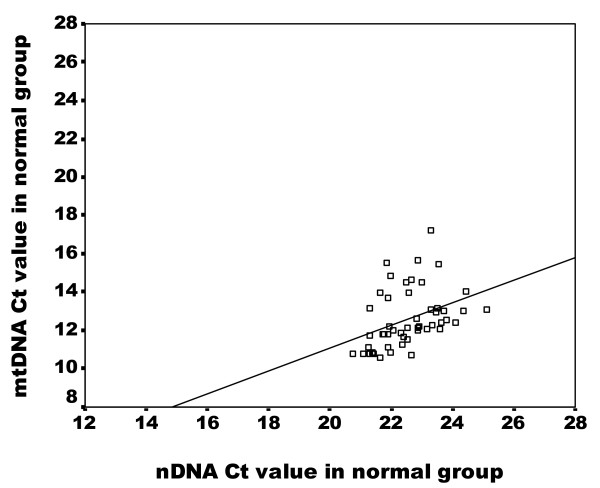
**Correlation of nDNA content and mtDNA content in peripheral blood in normal group**. Scatter plot illustrates the Ct values of GAPDH amplicon (X axis) against the Ct values of MTATP 8 amplicon (Y axis) in blood samples of normal group (n = 50). The correlation rate is highly significant (p = 0.0001) according the Spearman' rho test.

**Figure 2:**
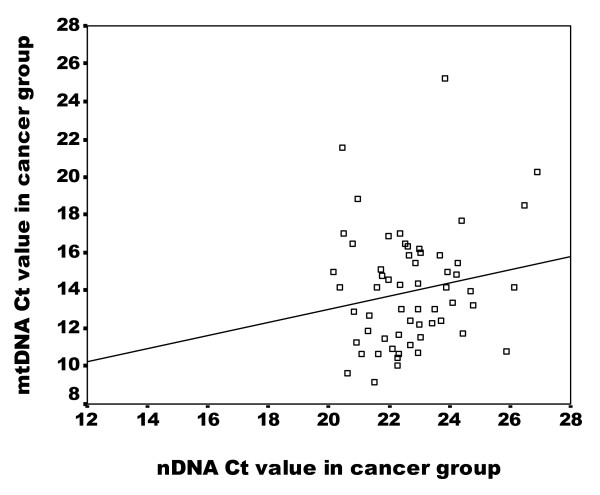
**Correlation of nDNA content and mtDNA content in peripheral blood in breast cancer group**. Scatter plot illustrates the Ct values of GAPDH amplicon (X axis) against the Ct values of MTATP 8 amplicon (Y axis) in blood samples of breast cancer group (n = 60). The correlation rate is not significant (p = 0.259) according the Spearman' rho test.

The average Ct values of GAPDH amplicon in the normal group were correlated with those of MTATP8 gene amplicon (Spearman's rho Test: p < 0.001, r = 0.496) (Figure 1). In contrast, no significant correlation was detected in cancerous group (p > 0.05, r = 0.148) (Figure 2), suggesting an alteration of the relationship between nDNA and mtDNA in blood samples of breast cancer patients.

### mtDNA in whole blood samples from the normal and breast cancer group

We used a formula of 2^ΔCt ^to calculate the content of mtDNA in whole blood. The area under the ROC curve for the mtDNA content was 0.76 (95% confidence interval, 0.67-0.87) with sensitivity of 73 and specificity of 78%. A 10.43 cycle difference in normal group and a 9.36 cycle difference in cancerous group were found by calculating the ΔCt values between GAPDH amplicon and MTATP 8 gene amplicon. The ΔCt values between normal and cancerous group showed a 1.07 cycle difference (ΔΔCt) (p < 0.01) as shown in Table 2.

### Relationship between decreased content of mtDNA and clinical parameters

**Figure 3:**
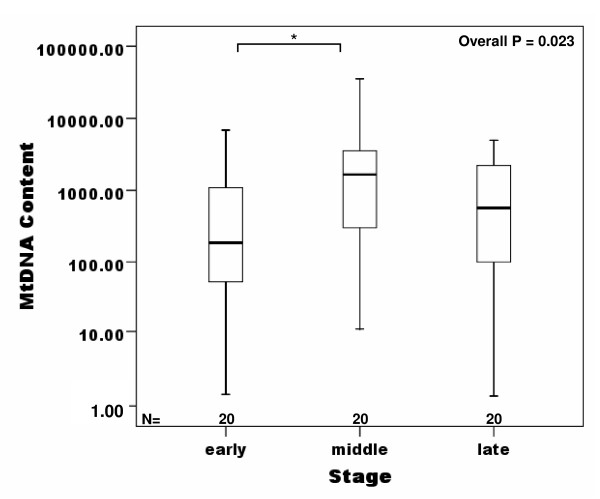
**Box plot analysis illustrating levels of mtDNA in peripheral blood of normal group and breast cancer groups according the stage ofcarcinomas**. The quantitative mtDNA content (as described in the text) is shown on the Y axis. The mtDNA content in peripheral blood of stage I breast cancers is significant lower than in the other stages according the One-way ANOVA on the ranks (*P = 0.018). Horizontal lines: group medians; boxes: 25--75% quartiles,, range, peak and minimum.

**Figure 4:**
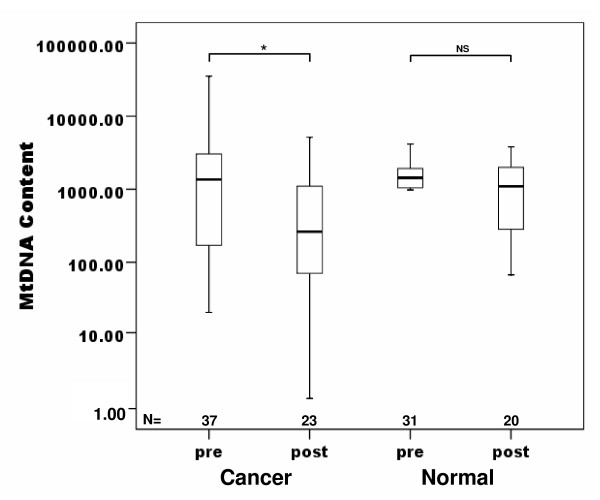
**Box plot analysis illustrating levels of mtDNA in peripheral blood of breast cancer patients according the menopausal status**. The quantitative mtDNA content is shown on the Y axis. The mtDNA content in peripheral blood of postmenopausal patients is significant lower than premenopausal patients according the Mann-Whitney U Test (* P = 0.024, NS: not significant); Horizontal lines: group medians; boxes: 25--75% quartiles, range, peak and minimum.

mtDNA content was compared with various clinical and pathological parameters of breast cancer patients such as age, menopause, TNM stage, lymph node status, ER-, PR- and Her-2/neu expression (Table 1). No significant difference in mtDNA content of peripheral blood was observed in regards to age, lymph node involvement, ER-, PR- and Her-2/neu expression (Table 1). However, the mtDNA content was significantly lower in stage ^2 ^than in the other stages (Table 1 and Figure 3, overall P = 0.023, I *vs *II, P = 0.018). The decreased mtDNA content in breast cancer patients was also correlated to menopausal status of beast cancer patients (Table 3 and Figure 4, P = 0.024). Significant decreased mtDNA content of peripheral blood was found in postmenopausal patients with cancer compared with that in postmenopausal normal group (Figure 4, P = 0.019).

**Table 3:** The correlation between the menopausal status and the levels of mtDNA in peripheral blood of breast cancer and normal groups

Variable	Number	mtDNA content Median (Range)	P value
Cancer			
Menopausal status			
pre	37	1365.30 (0.47-35241.80)	0.024
post	23	262.29 (0.39-5166.60)	
Normal			
Menopausal status			
pre	31	1443.15 (136.71-4182.07)	0.396
post	20	1097.52 (66.95-3808.48)	

P values are obtained by using the Mann-Whitney U Test.

## Discussion

mtDNA is vulnerable to DNA damage due to: a) the lack of protective histones; b) insufficient DNA repair capacity; c) a high rate of reactive oxygen species generation in mitochondria [22]. Mitochondria play an essential role in cellular energy metabolism, free radical generation and programmed cell death [23]. According to these characteristics and functions of mtDNA, it has been noted that alterations found in mtDNA could be involved at the early stage of carcinogenesis [24] and may play an important role in mitochondrial dysregulation and cancer development [9]. In addition, previous studies have suggested the potential use of mtDNA as biomarker for the early detection of cancer [25]. Low mtDNA content was identified in tumors specimens of breast cancer at an early stage, suggesting that it might be essential in early progression of breast cancer, most probably by impairing the oxidative phosphorylation or mitochondrial bioenergetics [8]. Jiang et al. showed a significantly increased mtDNA content in the saliva with advanced stage [15]. The change of mtDNA may therefore be important for cancer initiation and progression [14]. However, few studies have addressed the deletion and mutation of mtDNA in tumor tissues and blood of patients with breast cancer and the published results were inconsistent. Recent studies did not detect mtDNA mutations and deletions in the blood of women with breast cancer, implying that the mtDNA deletion and mutation play a minor role in breast carcinogenesis [26-28]. Furthermore, no previous data exist regarding the content of mtDNA in peripheral blood of breast cancer patients. In our study, we used MTATP 8 gene, a mitochondrial DNA encoded ATPase, to analyze the quantities of mtDNA in peripheral blood. We show that mtDNA content in whole blood from breast cancer patients significantly decreased in stage I breast cancer samples as compared with more advanced stages. This suggests that breast cancer cells depleted a high amount of mtDNA in the circulating system. mtDNA depletion could affect progression and metastasis of cancer cells by preventing apoptosis and generating cancer-related proteins [22]. The high frequency of mtDNA alterations in cancer and their presence in the stages I disease could possibly be exploited as clinical markers for early cancer detection [29]. Interestingly, Barrientos et al. reported that an increase in mtDNA content might be a compensatory response for decline in respiratory function [30]. So, that was why our results showed that mtDNA content firstly decreased at stage I and subsequently increased by the normal levels at stage II and stage III-IV.

A previous quantitative analysis suggested that the mtDNA content did not change with age of patients [31]. Similarly, our results displayed no significant difference between the decreased mtDNA content and age of patients. However, a recent report showed that the reduced copy number in mtDNA was more frequent in patients older than 50 years of age [8]. The relationship, therefore, between mtDNA content and patient's age needs further investigation. mtDNA circulates in peripheral blood not only in a cell-free form but also in a particle-associated form [32]. Mehra et al. showed that circulating concentrations of genomic DNA and mtDNA were not correlated with each other in plasma, probably due to the different compartmentalization and degradability of mtDNA and nDNA in cancers [33]. In line to this report, our results revealed no significant association between mtDNA content and nDNA content in peripheral blood from breast cancer patients.

The association between mtDNA content in peripheral blood and patients' age or clinical-pathological parameters was determined. No correlations between mtDNA content in peripheral blood and those parameters of breast cancer could be observed. Yu et al. have previously shown reduced mtDNA content to have a significant correlation with ER or PR status [8,19]. Endocrine treatment is essential for premenopausal as well as postmenopausal breast cancer patients [34]. Several studies showed that mitochondria respiration varies during the menstrual cycle and is correlated with pregnancy[35,36]. In our study, reduced mtDNA content inclined to have a significant correlation with menopausal status of breast cancer patients. However, whether reduced mtDNA in peripheral blood of breast cancer patients could potentially affect the outcome of endocrine treatment of breast cancer remains unknown.

## Conclusion

Our data suggest that the decrease of mtDNA content in peripheral blood of patients with breast cancer may have diagnostic and prognostic value. Decreased mtDNA content is associated with the menopausal status of patients and TNM stage of cancers. The use of mtDNA may have diagnostic and prognostic value and further studies are required to validate it as a potential biomarker for early detection of breast cancer.

## Competing interests

The authors declare that they have no competing interests.

## Authors' contributions

HXA, XYZ, WH, RE-C and CXD conceived of the study and participated in its design and coordination. PX participated in DNA extraction and real time PCR experiment, carried out the relevant statistical analysis, and drafted the manuscript. RR, PX and CK collected clinical data. HXA and EF helped to draft the manuscript. All authors read and approved the final manuscript.

## Pre-publication history

The pre-publication history for this paper can be accessed here:

http://www.biomedcentral.com/1471-2407/9/454/prepub
